# RrWRKY1, a Transcription Factor, Is Involved in the Regulation of the Salt Stress Response in *Rosa rugosa*

**DOI:** 10.3390/plants13212973

**Published:** 2024-10-24

**Authors:** Fengqi Zang, Qichao Wu, Zhe Li, Ling Li, Xiaoman Xie, Boqiang Tong, Shuhan Yu, Zhaoan Liang, Chunxue Chu, Dekui Zang, Yan Ma

**Affiliations:** 1 Key Laboratory of State Forestry Administration for Silviculture of the Lower Yellow River, College of Forestry, Shandong Agricultural University, Tai’an 271018, China; 2 Shandong Provincial Center of Forest and Grass Germplasm Resources, Jinan 250102, China

**Keywords:** WRKY transcription factors, abiotic stress, salinity tolerance, *Rosa rugosa*

## Abstract

Salt stress has become a major environmental problem affecting plant growth and development. Some WRKY transcription factors have been reported to be involved in the salt stress response in plants. However, there are few studies on the involvement of WRKYs in the salt stress response in *Rosa rugosa*. In this study, we isolated a salt tolerance gene, *RrWRKY1*, from *R. rugosa*. RrWRKY1 was found to belong to Group I of the WRKY family, and it was specifically expressed in leaves and petals. *RrWRKY1* expression was upregulated under NaCl stress in rose leaves. After silencing *RrWRKY1* in *R. rugosa*, transgenic plants showed dry leaves and black and brown veins, indicating sensitivity to salt stress. At the same time, the transcription levels of the salt tolerance-related genes *RrNHX1*, *RrABF2*, *RrRD22*, *RrNCED1*, and *RrHKT1* also changed significantly. The superoxide dismutase (SOD) and peroxidase (POD) activities decreased, the proline content decreased, and the malondialdehyde (MDA) content in the gene-silenced plants increased, indicating that *RrWRKY1* regulates the salt tolerance of *R. rugosa.* In addition, the overexpression of *RrWRKY1 in Arabidopsis thaliana* improved the germination rate and the average of the main root and lateral root lengths, and the transgenic plants had a larger number of lateral roots than the WT plants under salt stress. This study provides candidate gene resources for salinity tolerance breeding and a theoretical basis for analyzing the salinity tolerance mechanism of the *WRKY* gene.

## 1. Introduction

At present, more than 100 countries in the world have different degrees of soil salinization, among which the saline–alkali area in Asia is the most serious, with the saline–alkali land area in China being relatively large [1,2]. Salt stress leads to a severe imbalance in osmotic pressure inside and outside cells [3] and an imbalance in the Na^+^/K^+^ ion ratio in plants, which inhibits cell metabolic activity and may lead to plant wilting and death [4,5]. In addition, the increase in ROS (reactive oxygen species) caused by salt stress damages plasma membranes, affecting the transport of nutrients in plants, inhibiting normal growth, and even causing death in serious cases [6]. Therefore, it is of great significance to explore salt-tolerant germplasm resources and the function of salt-tolerance genes. *Rosa rugosa* is a deciduous shrub originating from the coastal areas of Jilin, Liaoning, Shandong Province in China, where land salinization is quite serious. Due to its strong tolerance, it is an excellent salt-tolerant germplasm resource that is of great significance and excellent for studying the salt tolerance mechanism [7].

In order to resist stress, plants have established complex mechanisms to adapt to various adverse environments through multiple signaling pathways during evolution [8]. The WRKY transcription factor family is one of the largest transcription factor families, and it plays indispensable roles in plant growth and development as well as participating in biotic and abiotic stress processes. The WRKY protein family can be divided into three different groups: Group I has two WRKY domains, Group II has one WRKY domain containing the same Cys2-HIS2 zinc finger motif, and Group III has one WRKY region containing different Cys2-HUS2 zinc finger motifs [9]. The WRKY transcription factors (TFs) are involved in the signaling pathways of various plant hormones, such as gibberellin (GA) and abscisic acid (ABA), thereby regulating various physiological processes in plants [10,11]. In previous studies, many *WRKY* genes were found to respond to salt stress. *Chenopodium quinoa* contained 92 *CqWRKY*s, and more than 50% were induced to be expressed under salt or drought stress [12]. A large number of experiments have shown that the WRKY family protects plants from salt stress by regulating plant physiological changes and phenotypes, such as superoxide dismutase (SOD), peroxidase (POD), photosynthetic efficiency, the germination rate, the root length, and the plant fresh weight [13]. The overexpression of the *Gossypium hirsutum GhWRKY34* in *Arabidopsis thaliana* showed that GhWRKY34, as a positive transcriptional regulator, may play a role in the plant response to high salt stress by maintaining Na^+^/K^+^ homeostasis and activating genes related to salt stress in cells [14]. *Malus domestica* MdWRKY31 was found to be a positive regulator of ABA signaling, and the overexpression of *MdWRKY31* in roots and calli showed a high level of sensitivity to ABA [15]. Several *AtWRKY*s were found to be involved in the ABA signaling pathway, thereby regulating salt stress in *A. thaliana* [16].

Through our previous study, we obtained the genome of *R. rugosa* [17], and a large number of *WRKY* genes were found in the genome and transcriptome data. Among the *WRKY* family, *WRKY1* has been reported to play a role in the stress response and growth and development of several species. In *G. barbadense*, *GbWRKY1* acted as a negative regulator of ABA signaling via *JAZ1* and *ABI1*, with effects on salt and drought tolerance [18]. In *A. thaliana*, *WRKY1* promoted the transition from vegetative to reproductive stages by inhibiting *FLC* expression as well as positively regulating SA biosynthesis genes to accelerate leaf senescence [19]. *NbWRKY1* positively regulated CCDaV-RepA-induced cell death in *Nicotiana benthamiana* [20]. In *Populus euphrysticum*, *PeHSF* bound to the cis-acting element of the *PeWRKY1* promoter enhanced the promoter activity and enhanced salt tolerance [21]. *HIS1-3* and *WRKY1* oppositely modulated salt tolerance in *A. thaliana* through the transcriptional regulation of SOS genes [22]. However, there has been little study of the function of *WRKY1* in *R. rugosa*. Therefore, we attempted to explore the role of *RrWRKY1* under salt stress through gene silencing in *R. rugosa* and overexpression in *A. thaliana*.

## 2. Results

### 2.1. Isolation and Sequence Analysis of RrWRKY1

**Figure 1:**
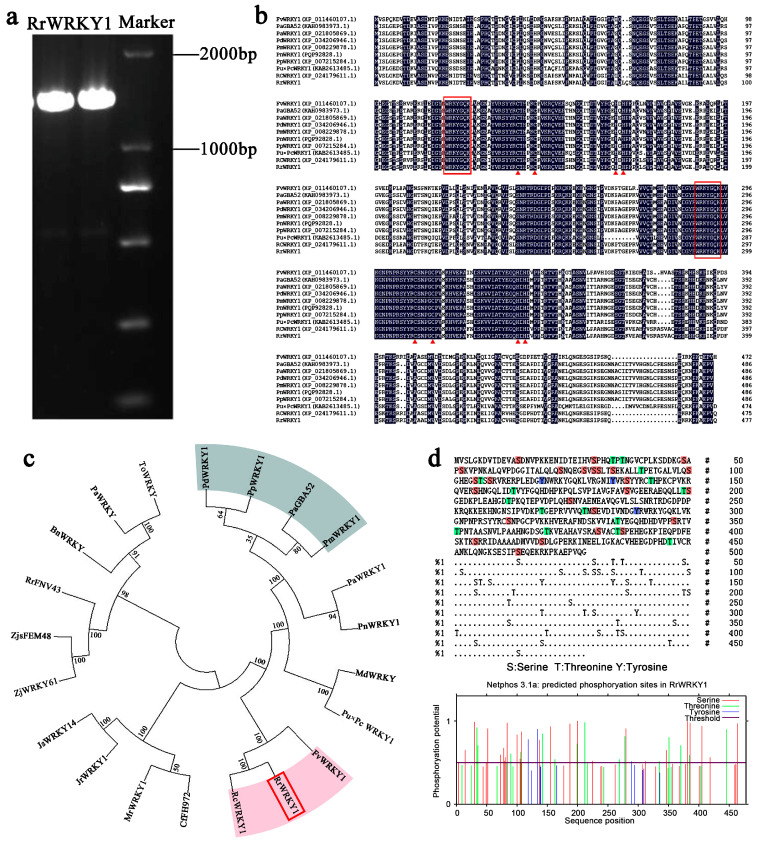
Cloning and bioinformatics analysis of *RrWRKY1*. (**a**) PCR amplification products of *RrWRKY1*. Marker: DNA marker 2000. (**b**) A conversed domain analysis of RrWRKY1 and its homologs in other plants. The red box indicates the heptapeptide sequence of WRKYGQK, and the red triangle indicates the zinc finger structure. (**c**) A phylogenetic tree of proteins encoded by RrWRKY1 and WRKY1 in other species. The red box indicates RrWRKY1. (**d**) Prediction of the phosphorylation sites of the RrWRKY1 protein. S-Ser; T-Thr; Y-Tyr.

Among the *WRKY* genes in the *R. rugosa* genome, we obtained a gene named *RrWRKY1* (OM038685). The cDNA length of the *RrWRKY1* amplification product was 1437 bp (Figure 1a), encoding 478 amino acids, and it contained two conserved WRKYGQK domains (pfam03106), indicating that RrWRKY1 belongs to Group I of the WRKY family.

The amino acid sequences encoded by *RrWRKY1* and its homologs in other plants were aligned (Figure 1b). Nine amino acid sequences with high homology to RrWRKY1 were selected for multiple sequence alignment. Like the other WRKY1 proteins, two WRKY domains in the RrWRKY1 coding region were found. The first WRKY domain contained 57 amino acids (positions 115–171), and the second WRKY domain contained 58 amino acids (positions 285–342).

The WRKY1 protein sequences of 21 different species were selected to construct a phylogenetic tree using MEGA7.0 with the neighbor-joining method. RrWRKY1 was the most closely related to *R. chinensis* RcWRKY1, with 100% homology originating from the same branch, followed by *Fragaria vesca*. In contrast, *Prunus persica*, *P. dulcis*, *P. avium*, and *P. mume*, which clustered into a clade, were more distantly related to *R. rugosa*, with an identity of 35% (Figure 1c). The molecular formula of the predicted RrWRKY1 protein was C_2245_H_3603_N_679_O_737_S_11_, the relative molecular mass was 52.251 kDa, and the total atomic number was 7275. Multiple phosphorylation sites were identified in the RrWRKY1 protein, indicating that reversible phosphorylation regulation plays an important role in realizing its function (Figure 1d).

### 2.2. Expression Patterns of RrWRKY1 in R. rugosa

**Figure 2:**
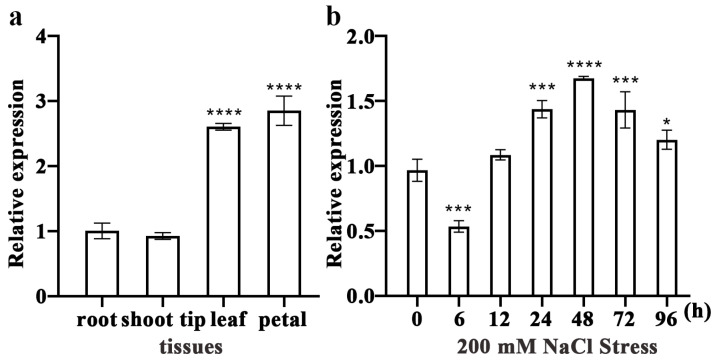
Relative expression analysis of *RrWRKY1* in *R. rugosa.* (**a**) Expression levels of *RrWRKY1* in different *R. rugosa* tissues. (**b**) Expression levels of *RrWRKY1* in leaves of *R. rugosa* under 200 mM NaCl salt treatment for 0 h–96 h. Asterisks indicate a significant difference between treatment and control: * *p* < 0.05; *** *p* ≤ 0.001; **** *p* ≤ 0.0001.

The expression levels of *RrWRKY1* in different tissues and organs were evaluated using qRT-PCR. We found that it was expressed in the roots, shoot tips, leaves, and petals, and the expression levels significantly differed among the tissues. The expression of *RrWRKY1* was higher in the leaves and petals and lower in the shoot tips (Figure 2a).

We analyzed the expression levels of *RrWRKY1* in leaves under 200 mM NaCl stress. Within 96 h under salt stress, *RrWRKY1* reached its peak expression at 48 h, and its expression gradually decreased with time (Figure 2b). After 96 h of salt stress, the expression level of *RrWRKY1* was still higher than that of the control. The expression level of *RrWRKY1* changed most obviously at approximately 48 h, while the changes were stable at the other times. These findings indicate that *RrWRKY1* may be involved in the regulation of the salt stress response.

### 2.3. RrWRKY1 Silencing Reduced the Tolerance of R. rugosa to Salt Stress

**Figure 3:**
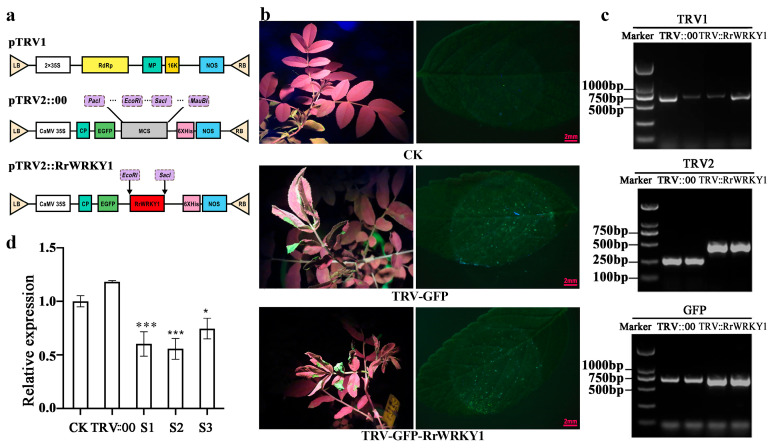
Detection of *RrWRKY1* gene silencing in *R. rugosa*. (**a**) Schematic diagram of the pTRV1 and pTRV2 plasmids and *RrWRKY1* target site designed in TRV2. LB: left border; RB: right border; RdRp: RNA-dependent RNA polymerase; MP: movement protein; 16k: 16kD protein; NOS: NOS terminator; CP: coat protein; MCS: multiple cloning site; EGFP: GFP. (**b**) Verification of GFP fluorescence in new leaves of *RrWRKY1*-silenced plants using excitation light source and fluorescence stereomicroscope. CK: control; TRV-GFP and TRV-GFP-RrWRKY1: VIGS-treated groups. (**c**) PCR detection of TRV1, TRV2, and GFP in leaves. TRV::00: TRV-GFP; TRV::RrWRKY1: TRV-GFP-RrWRKY1. (**d**) *RrWRKY1* gene expression levels in leaves of control and VIGS-treated groups. S1, S2, and S3 are three independent *RrWRKY1* gene-silenced plants. Asterisks indicate a significant difference between treatment and control: * *p* < 0.05; *** *p* ≤ 0.001.

To determine the function of *RrWRKY1*, the transcript level of *RrWRKY1* was knocked down in rugose rose plants using a virus-induced gene silencing (VIGS) approach. A pTRV2-*RrWRKY1* vector was constructed using a 286 bp fragment of *RrWRKY1* with EcoRI and SacI restriction sites (Figure 3a). After 14 days of infection, the newly grown leaves of the VIGS-treated groups (TRV-GFP and TRV-GFP-*RrWRKY1*) and the control group (CK) were tested for GFP green fluorescence. The GFP images showed that the leaves treated with VIGS showed green fluorescence under longwave ultraviolet light and a fluorescence stereomicroscope, while the untreated leaves in the control group showed red fluorescence (Figure 3b). The TRV1, TRV2, and GFP bands could be amplified in all the VIGS-treated groups, which proved that TRV::00 and TRV::*RrWRKY1* successfully infected *R. rugosa* and were expressed (Figure 3c). Furthermore, the corresponding leaves were collected for qRT-PCR to detect the silencing efficiency of *RrWRKY1,* using the *RrGAPDH* gene as an internal control. The expression levels of *RrWRKY1* in the *RrWRKY1* silencing groups (TRV::*RrWRKY1*) were significantly lower than those in the control group and TRV::00, indicating that *RrWRKY1* was effectively silenced in the rose, with the silencing efficiency being approximately 40% (Figure 3d). The silencing efficiency in the present study is similar to that of *RrGT2* in *R. rugosa* leaves in a study by Sui et al.; thus, it could be concluded that *RrWRKY1* was effectively silenced in *R. rugosa* leaves [23].

**Figure 4:**
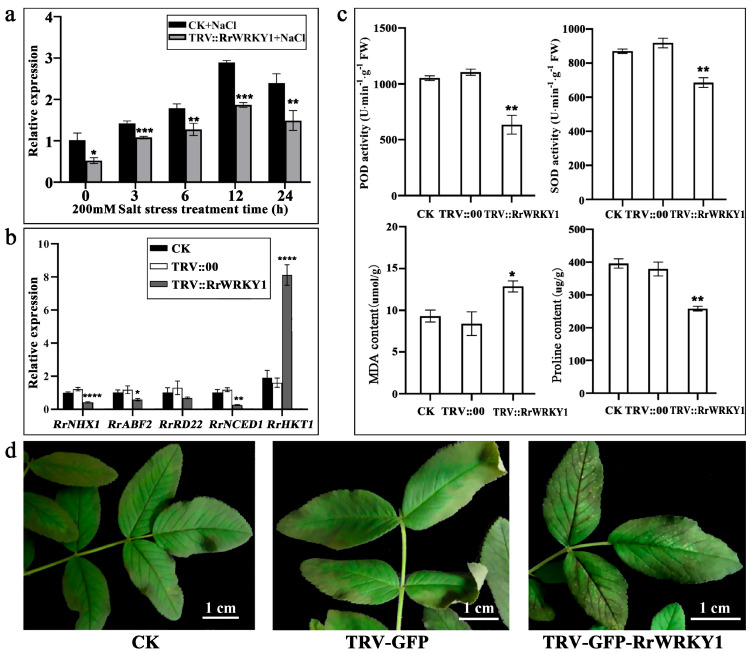
*RrWRKY1* silencing reduced the tolerance of *R. rugosa* to salt stress. (**a**) Expression analysis of *RrWRKY1* in leaves before and after *RrWRKY1* silencing under salt stress. (**b**) Comparison of the expression levels of salt stress-related genes in the *RrWRKY1*-silenced and control groups under salt stress. (**c**) Physiological index analysis between the control and VIGS-treated groups under salt stress for 2 days. (**d**) Phenotypes of the control and VIGS-treated groups under salt stress for 60 h. Asterisks indicate a significant difference between treatment and control: * *p* < 0.05; ** *p* < 0.01; *** *p* ≤ 0.001; **** *p* ≤ 0.0001.

After 18 days of infection, the shoots of normal and gene-silenced *R. rugosa* plants were selected. The results showed that the relative expression of *RrWRKY1* in the *RrWRKY1*-silenced rugose rose plants was significantly lower than that in the CK under 0–24 h salt stress; however, the overall expression trend of *RrWRKY1* was consistent with that of the control group, and the repressed *RrWRKY1* still responded to salt stress (Figure 4a).

The expression levels of the *RrNHX1*, *RrABF2*, *RrRD22*, and *RrNCED1* genes in the silenced plants were significantly lower than those in the CK and TRV::00 groups, but the expression level of the *RrHKT1* gene was significantly higher, suggesting that *RrWRKY1* is involved in the regulation of salt tolerance in *R. rugosa*. The expression of the *RrNHX1*, *RrABF2*, *RrRD22*, *RrNCED1*, and *RrHKT1* genes may be regulated to improve the salt tolerance of *R. rugosa* (Figure 4b).

After 2 days of salt stress treatment, leaves were taken to analyze the changes in physiological activities in both the control and VIGS-treated groups. The results showed (Figure 4c) that the SOD activity, POD activity, and proline content in the silenced group significantly decreased compared with those in the CK and TRV::00 groups, while the MDA content was expressively increased. As seen from the changes in the physiological activities, with the decrease in *RrWRKY1*, stress-related enzyme activities were also weakened. The salt tolerance of *R. rugosa* was positively correlated with SOD and POD enzyme activities and the proline content but negatively correlated with the MDA content.

After 60 h of salt stress, the isolated shoots of the *RrWRKY1*-silenced rugose rose plants were more sensitive to salt stress than those of the control and TRV::00 groups. The leaves of the silenced group were more seriously affected by salt stress, and the veins of the *RrWRKY1*-silenced branches were dark brown with a prominent loss of green, while the control and empty carrier groups had mild stress symptoms (Figure 4d). These phenotypes demonstrate that *RrWRKY1* is involved in the resistance to salt stress and positively regulates salt tolerance.

### 2.4. Overexpression of RrWRKY1 Enhanced Salt Tolerance in Transgenic Arabidopsis thaliana

**Figure 5:**
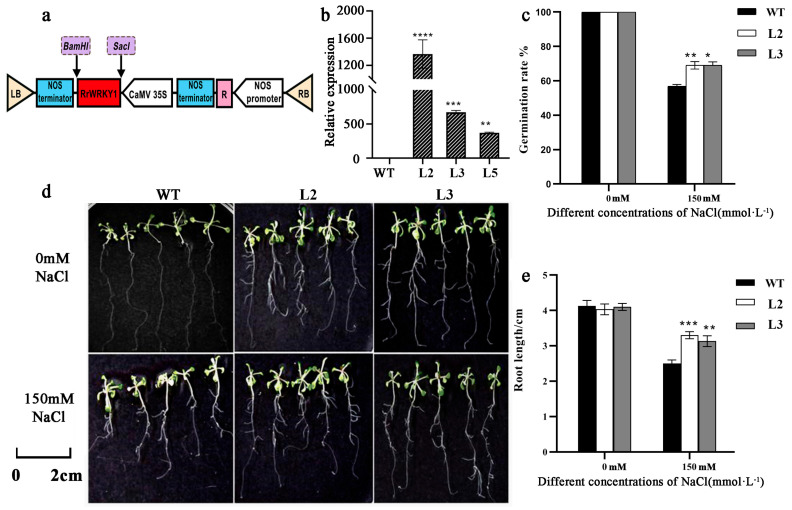
The heterologous expression of *RrWRKY1* improved salt tolerance in transgenic *Arabidopsis*. (**a**) Schematic diagram of the PBI121-*RrWRKY1* recombinant plasmid construction. (**b**) Expression levels of *RrWRKY1* in WT and transgenic *A. thaliana*. WT: Col-0; L2, L3, and L5, three independent transgenic *A. thaliana* lines. No transcripts of *RrWRKY1* were detected in WT. (**c**) Germination rate comparison of WT and transgenic *A. thaliana* cultured for 7 d on MS solid medium with 0 or 150 mM NaCl. (**d**,**e**) Root length comparison of WT and transgenic *A. thaliana* cultured for 20 d on MS solid medium with 0 or 150 mM NaCl. Asterisks indicate a significant difference between treatment and control: * *p* < 0.05; ** *p* < 0.01; *** *p* ≤ 0.001; **** *p* ≤ 0.0001.

Due to the short silencing time of TRV-VIGs, *RrWRKY1* was overexpressed in *A. thaliana* to further verify its function. The coding region of the *RrWRKY1* gene was expressed and transformed into *A. thaliana* to obtain transgenic composite plants, while the Col-0 wild-type (WT) was used as a control (Figure 5a). Three lines were selected to test the expression of *RrWRKY1* in the transgenic *A. thaliana*. It was found that two *A. thaliana* overexpression lines (L2 and L3) expressed *RrWRKY1* at significantly higher levels than the WT, so they were used for further examination (Figure 5b).

The WT and transgenic *A. thaliana* seeds were germinated on MS solid medium containing 0 and 150 mM NaCl. Under normal conditions (without NaCl), the germination rates of these two *A. thaliana* seeds were both 100%, while under 150 mM NaCl stress, the germination rates of both decreased, but the germination rates of the transgenic *A. thaliana* seeds were significantly higher than those of the WT. The germination rate of the transgenic *RrWRKY1* was 69%, while the germination rate of the WT was only 55% (Figure 5c).

The root lengths of the WT and transgenic *A. thaliana* were measured after 20 days of culture. Under 150 mM NaCl stress, the main root lengths of both decreased, but the lateral root length of transgenic *A. thaliana* was longer than that of the WT, and there were more lateral roots, indicating more development (Figure 5d,e). In conclusion, the overexpression of *RrWRKY1* can improve the salt tolerance of transgenic *A. thaliana* seeds.

## 3. Discussion

A series of complex physiological and biochemical processes occur in plants under abiotic stresses. For example, in a previous study, the overexpression of *VvWRKY13* in *Vitis vinifera* caused decreases in SOD, CAT, the photosynthetic rate, and the proline content and an increase in the oxygen free radical content; thus, it was found to be involved in salt stress tolerance through negative regulation [24]. The MDA content can be used to measure the degree of membrane lipid peroxide [25], which was found to inhibit the function of cell membrane proteins, leading to an impaired Na^+^ transport capacity and material leakage inside the cell and aggravated ion imbalance [26,27]. In this study, after *RrWRKY1* gene silencing, the activities of antioxidant enzymes and the content of proline decreased, while the content of MDA increased. This implies that the Na^+^ balance of rose leaf cells was disrupted by *RrWRKY1* silencing, which aggravated the damage caused by salt stress. Furthermore, *R. rugosa* showed phenotypic traits sensitive to salt stress after *RrWRKY1* silencing. The overexpression of *RrWRKY1* could improve the salt tolerance of *A. thaliana* seeds and seedlings. After stress, the main root elongation was inhibited in both the WT and *RrWRKY1*-overexpressed *Arabidopsis*, but the inhibition occurred to a lesser extent in overexpressed *Arabidopsis.* However, developments of lateral roots were observed in both the overexpressed and WT plants after 20 days of stress. Previous studies have shown that salt stress caused Root System Architecture (RSA) reprogramming and not only root development retardation [28]. High salt stress mainly restricted the growth and development of the primary root, while the lateral roots would undergo a period of developmental stagnation but would recover under long-term stress [29]. Similar results were obtained in our study, in which the *RrWRKY1* gene could enhance the development and growth of the *Arabidopsis* main root under a salt environment. On the other hand, the lateral roots of transgenic *Arabidopsis* were shown to be denser and stronger under non-stress conditions. Recent studies have shown that lateral root development and elongation can help plants remodel the root system under salt stress [29,30,31]. Based on the results of our experiments, we considered that *RrWRKY1* can help to respond positively to salt stress.

In addition, after *RrWRKY1* silencing, the expression levels of the *RrNHX1*, *RrABF2*, *RrRD22*, and *RrNCED1* genes, which are related to salt stress, were downregulated. A previous study showed that the overexpression of *MdNHX1* in *Malus* could increase K^+^/Na^+^ and thus enhance salt tolerance [32]. NCED is a key enzyme in ABA synthesis, and previous studies have proven that *NCED1* in *Citrus* can resist salt stress [33]. The *AtRD22* gene was first found to be regulated in *Arabidopsis* under salt, ABA, and drought stresses [34]. After soybean *GmRD22* was inserted into tobacco and *A. thaliana*, the salt tolerance of transgenic plants was significantly improved [35]. In response to stress, AREB/ABF received ABA signals, activated downstream stress-related genes, and regulated the interaction between functional proteins, thus putting plant cells in a balanced state [36]. In conclusion, the silencing of *RrWRKY1* reduced the expression of the above genes, which could positively regulate salt stress, further confirming the role of *RrWRKY1* in the regulation of salt stress.

Phosphorylation is the most extensive form of post-translational protein modification and the basis of life signal transduction [37]. Bioinformatics analysis showed that RrWRKY1 contained 64 Ser, 20 Thr, and 7 Tyr phosphorylation sites. Previous studies have shown that MAPK can directly phosphorylate WRKY and activate WRKY transcription factors, which are essential for the response of WRKY to different stresses [38]. AtWRKY53 can be directly phosphorylated by MEKK1 to enhance *AtWRKY53* expression. In addition, MEKK1 can directly phosphorylate the WP1 region on the *AtWRKY53* promoter to further promote its transcription [39]. These results suggest that the function of the RrWRKY1 protein may also depend on multiple phosphorylation sites.

The expression of *RrWRKY1* was higher in leaves and petals but lower in shoot tips and roots. In previous studies, tissue-specific expression patterns of *WRKY* genes were also observed in different plants [40]. In *A. thaliana*, *AtWRKY25* and *AtWRKY33* were found to be more abundantly expressed in roots than in leaves, stems, and seeds, and *AtWRKY33* transcripts were also more abundant in flowers [41]. When plants respond to salt stress, their various organs may have their own strategies. The expression of *RrWRKY1* was significantly higher in leaves than in other organs, so it was speculated that RrWRKY1 may play a role mainly in leaves. Therefore, we also selected leaves as experimental materials in gene-silencing experiments.

In a previous study, under salt stress, plant leaves sequestered Na^+^ in the vacuoles of epidermal or vascular bundle cells to alleviate Na^+^ damage to cytosolic enzymes [42]. In this regulation, the NHX family helped to absorb K^+^ on the vacuole membrane and regulate the accumulation of Na^+^ in order to reduce the concentration of Na^+^ [43,44]. Studies have shown that the transcription levels of *AeNHX1* and *AeNHX2* in *Agropyron elongatum* leaves were significantly higher than those in roots, suggesting that NHX1 and NHX2 can sequester Na^+^ in leaves under salt stress [45]. In this study, after silencing *RrWRKY1*, the expression of *RrNHX1* in leaves also decreased, indicating that *RrWRKY1* may affect the regulation based on *RrNHX1*, thereby enhancing the transport of Na^+^ in plant leaves to vacuoles.

It is worth noting that with *RrWRKY1* silencing, the expression of *RrHKT1* increased on the contrary. Extensive studies have shown that HKT maintained Na^+^ in the aboveground and underground parts of plants by promoting Na^+^ recycling in the phloem [46,47,48]. For example, AtHKT1 controlled root/shoot Na^+^ distribution and counteracted salt stress in leaves by reducing Na^+^ accumulation [49]. We hypothesized that a decreased expression of *RrWRKY1* reduced the Na^+^ transport to vacuoles, thereby increasing the Na^+^ concentration in leaves, which resulted in the upregulation of *RrHKT1* to promote downward Na^+^ transport. This phenomenon also implied that the salt stress response in *R. rugosa* involved multiple regulatory pathways.

## 4. Materials and Methods

### 4.1. Materials

The cuttings of 5-year-old *Rosa rugosa* were used for gene cloning and expression pattern analysis. One-year-old cuttings of *R. rugosa* were used for a functional analysis of gene silencing. The above-mentioned plant materials were all planted at the Forestry Experimental Station of Shandong Agricultural University in Taian City, Shandong Province, China.

Col-0 wild-type *Arabidopsis thaliana* (WT) was used for heterologous transformation to study gene function.

### 4.2. Gene Cloning and Phylogenetic Analysis

In a previous study, we obtained a large number of *WRKY* genes from *R. rugosa* genome data https://ngdc.cncb.ac.cn/bioproject/browse/PRJCA002607 (accessed on 5 February 2023), and *RrWRKY1* was obtained.

The total RNA was extracted from rose leaves using an Adlab EASY Spin Plant RNA Rapid Extraction Kit (Aidlab, Beijing, China). First-strand cDNA synthesis was performed according to the instructions of a Vazyme Reverse Transcription Kit (Vazyme, Nanjing, China). *RrWRKY1* was screened via the functional annotation of WRKY genes in the *R. rugosa* genome. Based on the open reading frame (ORF) sequence of *RrWRKY1*, we designed specific primers to clone the *RrWRKY1* CDSs (coding sequences). The primers are listed in Table S1.

The blast tool in NCBI https://blast.ncbi.nlm.nih.gov/Blast.cgi (accessed on 17 February 2023) was used to identify RrWRKY1 homologous sequences in other species, and DNAMAN 5.0 was used to translate target genes into protein sequences and perform a homologous protein sequence multiple comparison analysis. Nine sequences from other plants with high homology to the amino acid sequences of RrWRKY1 were selected for multiple sequence alignment. The species and GenBank numbers of these nine sequences are as follows: *Fragaria vesca* FvWRKY1 (XP_011460107.1), *Prunus avium* PaWRKY1 (XP_021805869.1), *Prunus armeniaca* PaGBA52 (KAH0983973.1), *Prunus dulcis* PdWRKY1 (XP_034206946.1), *Prunus yedoensis* var. *nudiflora* PnWRKY1 (PQP92828.1), *Prunus persica* PpWRKY1 (XP_007215284.1), *Prunus mume* PmWRKY1 (XP_008229878.1), *Pyrus ussuriensis × Pyrus communis* Pu × Pc WRKY1 (KAB2613485.1), and *Rosa chinensis* RcWRKY1 (XP_024179611.1).

The WRKY1 proteins of 21 different species were selected to construct a phylogenetic tree using MEGA7.0 [50]. The species and GenBank numbers of these 21 sequences are as follows: *R. rugosa* RrWRKY1 and *Malus domestica* MdWRKY (QDL95016.1), *Ziziphus jujuba* ZjWRKY1 (XP_015881045.1), *Z. jujuba* var. spinosa ZjsFEM48 (KAH7533461.1), *Rhamnella rubrinervis* RrFNV43 (KAF3443293.1), *Carpinus fangiana* CfFH972 (KAE8075540.1), *Morella rubra* MrWRKY1 (KAB1208675.1), *Parasponia andersonii* PaWRKY (PON63573.1), Trema orientale ToWRKY (PON88464.1), *Boehmeria nivea* BnWRKY (QQV37201.1), *Juglans regia* JrWRKY1 (XP_018824691.1), and *Juglans sigillata* JsWRKY14 (QWQ79436.1).

We analyzed the physical and chemical properties of the RrWRKY1 protein through the ExPasy ProtParam tool http://web.expasy.org/protparam/ (accessed on 9 March 2023) [51]. The online tool NetPhos 3.1 Serve https://services.healthtech.dtu.dk/service.php?Netphos-3.1/ (accessed on 9 March 2023) was used to predict the phosphorylation sites of RrWRKY1 [52].

### 4.3. Expression Pattern Analysis of RrWRKY1

For gene expression analysis of different tissues and organs, annual cuttings of *R. rugosa* with the same growth status, healthy growth, and no diseases or pests were selected and managed in pots. The roots, shoot tips, leaves, and petals of *R. rugosa* were collected at a height of 20–25 cm. For gene expression analysis under salt stress, a total of 18 pots of plants with the same size and growth status were treated with 200 mM NaCl. The control group was watered as usual (0 mM NaCl), and all the pots were slowly and evenly watered with 300 mL NaCl or water. The leaves of at least three plants were collected after 0, 6, 12, 24, 48, 72, and 96 h of treatment and then quickly frozen using liquid nitrogen and stored at −80 °C until further use.

The expression levels were measured using qRT-PCR. The template cDNA samples were set in triplicate and analyzed using the 2^−ΔΔCT^ method [53]. *RrGAPDH* was used as the reference gene [23], and fluorescent quantitative primers were designed according to the correct sequence sequenced, as shown in Table S1. The reaction conditions and procedure were performed according to the specifications of Vazyme ChamQ Universal SYBR qPCR Master Mix (Vazyme, Nanjing, China).

### 4.4. Silencing Experiment

According to the cloned CDS of the *RrWRKY1* gene, SnapGene was used to select the MCS restriction sites EcoRI and SacI on the pTRV-GFP empty vector, and a recombinant pTRV-GFP-*RrWRKY1* silencing vector was constructed by Tsingke Biotechnology Co., Ltd. (Tsingke, Beijing, China). The vector was introduced into the GV3101 *Agrobacterium tumefaciens*. Overnight-grown *A. tumefaciens* cultures were pelleted and resuspended in osmotic buffer (10 mM MES, 10 mM MgCl_2_, 150 μM AS, sterile water as solvent) to a final OD600 of 1.5. The resuspended solution with the same concentration was mixed with an equal volume of pTRV1 (1:1). To improve the efficiency of infection, the surfactant silwet L-77 (Coolaber, Beijing, China) was added to the infection solution after mixing to a final concentration of 0.01%, and the solution was left at 25 °C for 4 h in the dark to prepare for infection.

One-year cuttings of *R. rugosa* planted in pots containing peat soil were selected and densely scratched on the back of leaflets, the petiole of leaflets, and the base of compound leaves for infection. After one day under dark conditions, the plants were cultivated in a manual climatic box at 25 °C with 50% relative humidity, a 16 h/8 h day/night period, and the light intensity was around 150 μmol·m^−2^·s^−1^. After 14 days of infection, the infected site and the newly grown leaves on the top of *R. rugosa* were irradiated with a Luyor-3415RG excitation light source (Luyor, Shanghai, China), and the newly grown leaves were also observed using an SZX16 fluorescence stereomicroscope (Olympus, Tokyo, Japan) to detect whether the GFP was successfully transferred into *R. rugosa* [54]. The TRV1/TRV2/GFP bands were amplified to confirm the successful infection of the treated plants’ newly grown leaves. The efficiency of gene silencing was measured using qRT-PCR, using *RrGAPDH* as the reference gene. All the primers used in this study are listed in Table S1.

### 4.5. Expression Analysis of Genes Related to Salt Stress in Isolated Silenced Plants

After 18 days of infection, the new twigs above the normal and gene-silenced infection sites were cut, washed, and set in sterile deionized water for 2 h, and then they were transferred to conical flasks containing 0 and 200 mM NaCl solution for experimentation. The *R. rugosa* leaves of both groups were sampled after 0, 3, 6, 12, and 24 h. The expression of *RrWRKY1* before and after silencing was analyzed using qRT-PCR. The sequences of the *RrNHX1*, *RrABF2*, *RrRD22*, *RrNCED1*, and *RrHKT1* genes, which may be related to salt stress, were screened via sampling after 24 h of salt stress treatment. Specific primers were designed, and *RrGAPDH* was used as the reference gene to detect the expression trends of related genes after salt stress treatment in isolated and silenced *R. rugosa* using qRT-PCR. After 2 days of salt stress treatment, the SOD, POD, MDA, and proline contents in the leaves of the *R. rugosa* plants were measured [55,56]. After 60 h, the phenotypic changes in the *R. rugosa* leaves were observed and photographed.

The proline and MDA contents were determined using a Proline assay kit and a plant Malondialdehyde (MDA) assay kit, respectively. The SOD and POD activities were measured using a Total Superoxide Dismutase (T-SOD) assay kit and a Peroxidase assay kit, respectively. All the above-mentioned kits were bought from Beijing Biosynthesis Biotechnology Co., Ltd. (Beijing, China), and each physiological index was determined according to the manufacturer’s instructions.

### 4.6. Overexpression Experiment

The full-length *RrWRKY1* (1437 bp) was ligated with the linearized vector pBI121 to construct the recombinant plasmid PBI121-*RrWRKY1* and was then transformed into *Agrobacterium tumefaciens* GV3101; the primer sequences are shown in Table S1. Then, *RrWRKY1* was transferred to *Arabidopsis* via inflorescence impregnation, as described previously [57]. The harvested seeds were screened on MS plate medium containing 50 mg/mL kanamycin, and transgenic plants were obtained. Finally, homozygous line (T2 transgenic) seeds were obtained and continued to be seeded and grown in MS medium. After 7 days of germination, the transgenic seedlings were transplanted into a nutrient soil pot and grown at 22 °C under a 16 h/8 h light/dark period. Leaves from three independent lines of transgenic *Arabidopsis* were collected to extract the RNA using an Adlab EASY Spin Plant RNA Rapid Extraction Kit (Aidlab, Beijing, China). qRT-PCR was performed using Vazyme ChamQ Universal SYBR qPCR Master Mix (Vazyme, Nanjing, China). The internal control gene of *Arabidopsis* was *AtActin2*. qRT-PCR was repeated three times. The qRT-PCR primers are shown in Table S1.

### 4.7. Determination of Germination Rates and Root Length in Transgenic A. thaliana Under Salt Stress

After sterilizing the seeds of the WT and transgenic *A. thaliana* lines with a higher relative expression, they were further seeded on MS solid medium containing 0 and 150 mM NaCl, with 100 seeds per plate for 3 biological replicates. After 7 days, the total germination rates of each group were accurately calculated by region. Similarly, the WT and the same lines with a higher expression were seeded on normal MS plate medium and grown for 5 days, and then they were transferred to MS plate medium containing 0 and 150 mM NaCl and vertically grown for 15 days. Afterwards, the length of the main root was accurately measured. All of the above treatments were conducted in a light incubator at 22 °C under a 16 h light/8 h dark photoperiod, with 50–60% relative humidity.

### 4.8. Statistical Analysis

In this study, three biological replicates were analyzed for each treatment. All of the data were subjected to ANOVA using SPSS 20.0 (IBM, Inc., Armonk, NY, USA). The error bars represent the mean ± standard deviation of the data (±SD). The different asterisks indicate significant differences at *p* < 0.05, extremely significant differences at *p* < 0.01, etc.

## 5. Conclusions

We cloned a novel Group I *WRKY* gene, *RrWRKY1*, from *R. rugosa*, and the expression of this gene was induced by salt stress. In our study, the sensitivity of silenced *R. rugosa* to salt stress was enhanced. At the same time, the transcription levels of some salt tolerance-related genes also changed significantly. The ectopic overexpression of *RrWRKY1* increased the tolerance of transgenic *A. thaliana* to salt stress. This study identified a novel *WRKY* gene function that enhances tolerance to salt stress and is of great significance for understanding the regulation of salt stress in *R. rugosa*.

## Data Availability

All data and materials generated or analyzed during this study are included in this published article and its Supplementary Files. The sequence of *RrWRKY1* was uploaded to GenBank under accession number OM038685.

## Supplementary Materials

The following supporting information can be downloaded at https://www.mdpi.com/article/10.3390/plants13212973/s1, Table S1: All the primers used in this study.
